# Estimating South African Higher Education Productivity and Its Determinants Using Färe-Primont Index: Are Historically Disadvantaged Universities Catching Up?

**DOI:** 10.1007/s11162-022-09699-3

**Published:** 2022-05-27

**Authors:** Omphile Temoso, Lindikaya W. Myeki

**Affiliations:** 1 UNE Business School, University of New England, Armidale, NSW Australia; 2 Department Agricultural Economics, University of Free State, Bloemfontein, South Africa

**Keywords:** Total factor productivity, Higher education, Public universities, Färe-Primont index, University types, South Africa

## Abstract

Recent high dropout and low graduation rates in the South African higher education institutions as well as government funding cuts and the economic uncertainty due to COVID-19 pandemic have heightened the urgency for the higher education sector to improve its productivity. However, empirical evidence on the productivity growth of the sector remains unexplored. To address this gap, we applied a Färe-Primont index approach to a panel data of 22 public universities over an 8-year period to measure total factor productivity (TFP) and its components—technological change, technical, scale and mix efficiency changes. We also used a feasible generalised least squares model to assess the determinants of productivity and efficiency growth. The results show that the average TFP of the sector for the study period was 0.631, led by historically advantaged universities (0.894), whilst historically disadvantaged universities had lower average TFP (0.823). During the period, TFP increased by 3.43%, largely driven by scale and mix efficiency changes (5.32%) and technical efficiency change (0.83%), whilst technical change declined by 1.80%. In terms of university types, the comprehensive universities achieved the largest TFP growth (6.13%) followed by traditional universities (4.85%), and technology universities by 1.41%. TFP growth was positively influenced by student graduation rates, quality of academics and academic-student ratios. Therefore, policy considerations to improve the sector’s productivity and efficiency should consider investment on research and development, adoption of teaching and research innovations, re-skilling through training and education and aligning admission policies with staffing.

## Introduction

Over the past two decades many governments around the world have increasingly sought to maximise the ‘value for money’ from their investment in education (Blackburn et al., 2014). This need has led to many empirical studies focussing on measuring the efficiency and productivity of educational providers including higher education institutions (HEI) (Agasisti, 2017). Likewise in South Africa, the higher education sector is increasingly receiving more attention on this topic in relation to achievement of the country’s long-term development strategies (National Planning Commission [NPC], 2011). Therefore, there are three justifications that can be given for the quest to improve South Africa’s HEI productivity growth. Firstly, there are several government policies for the sector that include amongst others the *National Development Plan 2030 Strategy* (NPC, 2011)—which seeks to attain 75% of PhD qualified academic staff, 70% student participation rates at universities, 1.62 million student enrolment and train at least 100 PhD graduates per million people per year by 2030. These noble goals will depend on a better performing university sector in terms of productivity.

Secondly, the productivity of HEIs in South Africa is increasingly coming under serious scrutiny due to long-standing challenges faced by the sector such as the declining government grant funding, growing student enrolment rates (hence high student to staff ratios) and high dropout rates (Tjønneland, 2017). In fact evidence shows that despite significant efforts made by the South African democratic government through various policies to improve the efficiency, productivity and equity of the higher education sector and closing the gap between *traditionally disadvantaged* HEI (predominantly black, Indians and coloured universities) and *advantaged* HEI (predominantly white universities), some HEI in the country continue to perform poorly in terms of graduation rates and research output (Tjønneland, 2017; Department of Higher Education and Training [DHET], 2012). Moloi et al., (2014) estimates that on average only 35% of total student intake and 48% of contact students complete their study programs within the program duration. The Council of Higher Education (CHE, 2019) revealed that traditionally disadvantaged HEI are worse off than their peers in these statistics, thus, raising questions on whether policies aimed at closing the gap between traditionally advantaged and disadvantaged HEI are achieving their objectives. Hence, decision makers are interested in understanding the growth and determinants of productivity and efficiency of universities to identify areas for improvements.

Thirdly, given that public universities in South Africa receive most of their funding from the public coffers, there is increased attention paid to the performance—measured by efficiency and productivity, and public accountability for government funds devoted to higher education. According to the *Ministerial Statement on University Funding* “universities are required on an ongoing basis to practice efficiency measures and ensure that available funding is effectively utilised” (DHET, 2018a, b, p. 2). Some of the recommendations in the report include reducing overhead costs relative to the main functions of universities; improving debt collection; and seeking new ways to generate and source additional income funds (DHET, 2018a, b). Considering the impact of the COVID-19 pandemic on the government budget, which is expected to directly affect university funding in the foreseeable future, now more than ever before, enhancing universities efficiency and effectiveness has become more important. Accordingly, there is growing public demand for the universities to be accountable for the way they spend taxpayer’s money. Therefore, empirical evidence on the efficiency and productivity performance of universities is essential in providing insights for policy decisions and public confidence in the sector.

In the context of this study, we are interested in analysing the total factor productivity growth (TFP) for HEIs in South Africa. Our choice for this analysis is informed by the fact that TFP considers multiple inputs and outputs including their relationship and this approach has largely been ignored in the country’s literature. This implies that empirical evidence on HEI TFP growth, its components, and determinants in South Africa is scarce. For instance, research to date on South African HEI performance have largely focussed on equity and access (e.g., Badat, 2008; Boughey, 2003; Mabokela & Mlambo, 2017; Akoojee & Nkomo, 2007; Govinder et al., 2013) and technical efficiency (Marire, 2017; Myeki & Temoso, 2019; Nkohla et al., 2019; Taylor & Harris, 2004), whilst productivity and its sources including scale and mix (scope) efficiencies have not been explored.

Using data for 10 public universities for the period 1994 to 1997, Taylor and Harris (2004) found an average efficiency of 85% for institutions during the reported period. However, this study was limited to few universities and was performed using pre-university merger data and therefore doesn’t cover the higher education mergers implemented between 2002 and 2005. On the contrary, Marire (2017) used stochastic frontier to investigate cost efficiency of 22 universities in South Africa and estimated a mean efficiency of 87.3% indicating a potential to improve efficiency by 12.7%. However, the approach used in this study only accounts for single output variables and like Taylor and Harris (2004), the study is silent on the contribution of scale and mix efficiency to HEI productivity growth.

Recently, Myeki and Temoso (2019) and Nkohla et al. (2019) both applied DEA to estimate technical efficiency change for 22 South African universities over a period of 5 years (2009–2013), with the former concluding that more work still needs to be done to improve efficiency and the later that the performance benchmarks could be used as best practice to be emulated by inefficient institutions and proposed potential improvements required to reach a satisfactory level of efficiency. None of the studies have estimated TFP growth in the South African context.

This study aims to address this knowledge gap by measuring South African HEI productivity growth and its drivers over an 8-year period (2009 to 2016) using Färe–Primont index, a more recently developed TFP index by O’Donnell (2011). TFP analysis for the South African HEI provides an opportunity to compare the dynamics of productivity growth already established internationally (Carrington et al., 2018; Edvardsen et al., 2017; Flegg & Allen, 2007; Glass et al., 1998; Johnes, 2008; Miles et al., 2018) with the case of an economically developed African country. South Africa has some of the highly developed universities in the continent making them a popular destination for international students from across the continent (OECD, 2019). According to the OECD (2019) report, about 4% of the total tertiary student population in South Africa were from the neighbouring countries. To make South Africa one of the most competitive knowledge-based economies in the world, improving the productivity and efficiency of the sector would be critical.

This study contributes to the literature by measuring the efficiency and productivity growth of South African HEI while taking into consideration possible influences of economies of scope and scale which are critical for informing policies aimed at enhancing the productivity of the sector. Given that South African universities are diverse in terms of size and produce a variety of output mixes (e.g., undergraduate, masters, and PhD degrees as well as publications and patents, etc.), we argue that considering the potential contribution of scale and mix efficiency towards overall productivity is necessary. For example, the so-called traditional universities produce significant research and training outputs as well as a diverse range of graduates (bachelor, postgraduate and PhD graduates), whilst comprehensive and technology universities concentrate primarily on producing teaching outputs. These divergences can affect productivity and efficiency of the institutions in different ways.

From the best of our knowledge this is the first study to analyse total factor productivity growth in South African higher education, and by doing so, we add to the limited studies in this area in developing and emerging economies using advanced TFP index methods such as Färe–Primont index (Tran & Villano, 2017). Majority of existing studies in this area have largely focussed on developed economies (see, Witte & López-Torres, 2017 for review) and are limited to the application of Malmquist-based index approach (e.g., Agasisti & Johnes, 2009; Flegg & Allen, 2007; Worthington & Lee, 2008) and Törnqvist index approaches (e.g., Moore et al., 2019). However, as pointed out by O'Donnell (2011, 2012a, b, 2018), Malmquist and Törnqvist are not complete indexes because they do not account for the contribution of other efficiency measures such as scale and mix efficiencies towards TFP. On the other hand, Färe-Primont index satisfies all the established rules of TFP index numbers such as the identity and transitivity axioms (O’Donnell, 2017). Moreover, this index can decompose productivity into finer components of technical change, technical efficiency change, scale and mix efficiency changes, which permit for identification of different strategies to enhance productivity growth of the sector (Carrington et al., 2018; Temoso et al., 2015). Therefore, this is the first study to analyse productivity growth of South African HEI and its drivers using the most recently developed TFP index (Färe-Primont index).

In addition, we take advantage of the TFP index and its components and empirically test whether historically disadvantaged HEI are closing the performance gap with the historically advantaged universities. This allows us to assess whether performance gaps, in terms of productivity and efficiency changes, are widening or converging between the two groups.

The third contribution of this study is that it assesses the influence of institutional and regional characteristics on efficiency and productivity of universities using panel data models. None of the existing efficiency studies in South Africa (Myeki & Temoso, 2019; Marire, 2017; Nkohla et al., 2019; Taylor & Harris, 2004) have directly examined the predictors and factors influencing TFP and its components. Given that the cost of higher education is an important public policy issue, uncovering the predictors and institutional factors affecting productivity have potential to inform policies and decision makers in the higher education sector e.g., CHE which is accountable for funding universities through a ‘Block Grants’ system.^1^

The rest of the article continues as follows. Section “The South African University Sector” provides historical and policy overview of the South African university sector. Section Analytical Framework provides an overview on the empirical models, whilst Sect. “Data and Sources” provides details on data sources and variables. This is followed by results analysis and discussion in Sect. “Results and Discussion”. Finally, Sect. “Summary, Conclusions and Policy Implications” provides the conclusion.

## The South African University Sector

Several influential reports (CHE, 2004, 2007, 2016; Badat, 2008) have examined the evolution of South Africa's higher education sector, claiming that the majority of universities were established prior to the independence regime, beginning with the University of Cape Town, University of Witwatersrand, Stellenbosch University, Rhodes University, the University of Natal, the University of Potchefstroom, and the University of the Free State. Other institutions that came later included the University of Port Elizabeth (now Nelson Mandela University) and Rand Afrikaans University (now University of Johannesburg).

From the onset, these universities (except University of Fort Hare founded in 1916) were serving the interests of the white population with great reluctance to provide education for black people. This situation became worse with the enactment of the *Extension of University Education Act of 1959* promoting enrolment of Africans away from the white historically advantaged universities. Thus, leading to the establishment of historically disadvantaged universities which include amongst others the University of Western Cape, University of Fort Hare, University of Zulu Land, University of Bophuthatswana (now part of the University of North West) and University of Limpopo. As a result, towards the end of the apartheid regime in 1994, the higher education sector consisted of 21 public universities and 15 Technikons (Bunting, 2006; CHE, 2007) that were fragmented and uncoordinated. These inequalities are still deeply entrenched in the higher education system of the country. However, the democratic government has made significant effort to redress these inequalities through various policy instruments post-1994. These policies include amongst others the *1996 South African Constitution, South African Qualifications Authority *(*SAQA*)* Act of 1995*, *2001 National Plan for Higher Education*, *White Paper 3* of 1997, National Development Plan 2030 and *White Paper *(2014).

Despite these, much work is still required to improve the situation in these universities given the fact that they are increasingly under pressure to produce more outputs with less resources while redressing the institutional and structural inequalities for improved efficiency and productivity. However, to date the HEI sector in South Africa consists of 26 public universities classified into (i)* traditional universities* that offer basic formative degrees such as BA & BSc, and professional undergraduate degrees such as BSc Eng. and MBChB; at postgraduate level offer honours degrees, and a range of masters and doctoral degrees; (ii)* universities of technology* provide mainly vocational or career‐focused undergraduate diplomas and BTech which serves as a capping qualification for diploma graduates. It also offers a limited number of masters and doctoral programmes, and (iii)* comprehensive universities*—which offer programmes typical of the traditional university as well as programmes typical of the university of technology.

## Analytical Framework

### Estimating Universities TFP and Its Components Using Färe-Primont Index

Delivery of higher education involves the utilisation of valuable resources such as research and teaching inputs (e.g., student enrolments and availability of teaching and research staff) to produce the desired outputs such as graduates, research papers and patents. In this study, productivity refers to TFP which is described as the value of outputs produced for a given level of inputs over a given period (O’Donnell, 2017).

Following O’Donnell (2011) and Carrington et al. (2018), productivity growth of entities can be expressed in terms of aggregate output to aggregate input. Assuming *X*_*it*_ = (*X*_*1it*_*, …, X*_*Nit*_) is an input quantity vector and *Q*_*it*_ = (*Q*_*1it*_*, …, Q*_*Mit*_) is an output quantity vector for university *i* in period *t* then university TFP can be described as:

1 $${TFP}_{it}= \frac{{Q}_{it}}{{X}_{it}}$$

where TFP is the total factor productivity, *Q*_*it*_ = *Q*(*q*_*it*_) represent aggregate output whilst *X*_*it*_ = *X*(*x*_*it*_) represent aggregate input. *Q*(.) and *X*(.) are linearly homogeneous aggregator functions assumed to be non-negative and non-decreasing. An index number that estimates TFP of university *i* in period *t* relative to university *h* in period *s* can be presented as

2 $${TFP}_{hs,it}= \frac{{TFP}_{it}}{{TFP}_{hs}}= \frac{{Q}_{hs, it}}{{X}_{hs,it}}$$

where *Q*_*hs,it*_ = *Q*_*it*_*/Q*_*hs*_ representing an output quantity index, while *X*_*hs,it*_ = *X*_*it*_*/X*_*hs*_ represent an input quantity index. As a result, TFP is described as an estimation of output growth divided by input growth (O’Donnell, 2011). Then, O’Donnell (2011) shows that Eq. (2) can be decomposed into several components as follows:

3 $${TFP}_{hs,it}=\left(\frac{{TFP}_{t}^{*}}{{TFP}_{s}^{*}}\right)\left(\frac{{OTE}_{it}}{{OTE}_{hs}}\right)\left(\frac{{OSME}_{it}}{{OSME}_{hs}}\right)$$

where TFP_t_* is the maximum TFP possible given the existing technology in period t. OTE represents the Farrell output-orientated technical efficiency and OSME is a measured output scale mix efficiency. OSME measures potential gains from economies of scale and scope. A Data Envelopment Analysis (DEA) method is adopted to estimate the Färe-Primont index through the *DPIN 3.0* software (O’Donnell, 2011).

### Determinants of TFP Growth and Its Components Using Panel Data Models

Following the computation of TFP and efficiency indices, the next step is to understand how different university and regional characteristics have influenced the TFP and efficiency growth of universities. To achieve this, we used a feasible generalised least squares (FGLS) for panel data following other empirical studies on the determinants of TFP (e.g., Rahman & Salim, 2013; Widodo et al., 2014). FGLS is preferred over alternative panel data models such as fixed effects and random effects models because it allows us to estimate the structure of heteroskedasticity from OLS. Hence, it accounts for both systematic effect of universities and time varying effects of explanatory variables (Rahman & Salim, 2013). On the other hand, random and fixed effects commonly suffer from a variety of non-spherical error behaviours such as heteroskedastic.

The empirical model can be specified as follows:

4 $${y}_{kit}=\alpha +\beta {X}_{it}+{u}_{i}+{\varepsilon }_{it}$$

where ${y}_{k}$ represents the index of TFP change and/its components (k = 1, 2…0.4); X captures the matrix of explanatory variables (i.e. drivers of TFP and its components provided in the bottom part of Table 1 such as *academic-student ratio*, *proportion of academics with PhD*, etc.), $\beta$ is the vector parameters, ${u}_{i}$ represents unit specific random elements independent and identically distributed as IID (0, ${\sigma }_{u}^{2}$) and it is assumed to be independent from ${\varepsilon }_{it}$ and${x}_{it}$; and ${\varepsilon }_{it}$ is distributed and is assumed to be independent of *IID* (0, ${\sigma }_{\varepsilon }^{2})$. This model was estimated using STATA 15.

**Table 1:** Definition of variables in the model

Variables	Definition	Previous studies using similar variables
*Outputs*		
Undergraduates completed	Number of persons who have obtained a qualification with National Qualification Framework (NQF) exit levels 5, 6 and 7 as well as 1st bachelor’s degrees with NQF exit level 8	Most of the efficiency studies have included this variable as an output. Examples include Worthington and Lee (2008), Carrington et al. (2018), Johnes (2008)
Postgraduates completed	Number of persons who have obtained a qualification with NQF exit levels 8, 9 and 10 (excluding 1st bachelor degrees with NQF exit level 8)	This is also a commonly used variable in the literature, see for example, Myeki and Temoso (2019) and Worthington and Lee (2008)
Doctorates completed	Number of persons who have obtained a qualification which has a masters' degree as a minimum entry requirement	Other studies such as De Fraja and Valbonesi (2012), Kuah and Wong (2011), Worthington and Lee (2008) have also used this variable
Publications (research output)	Weighted sum of books, articles and conference papers published as well as the number of dissertations and thesis completed. For a given year, research masters graduates and publications are multiplied by 1 unit, while a doctoral graduate is multiplied by 3 units (DHET, 2019)	This is a similar variable used by Worthington and Lee (2008), Myeki and Temoso (2019) and Moore et al. (2019), and many other studies
*Block grants *(*South African Rands*)	This represents the total block grant received by a university in each period. The block grants are linked to performance and have four components: (1) teaching input which is based on enrolments, (2) teaching output which is based on graduations, (3) research output, based on approved publications and research students’ graduations, and (4) institutional factors based on size and proportion of historically disadvantaged students	Other studies including Kuah and Wong (2011), Cantele et al. (2016) used this variable to proxy research income
*Inputs*		
Undergraduates enrolled	Number of persons who are registered for a qualification with NQF exit levels 5, 6 and 7 as well as 1st Bachelor degrees with NQF exit level 8. It is measured using total headcount enrolment which implies that both full-time and part‐time students are considered as single units, irrespective of the course load each is taking	This is a commonly used variable in the HEI efficiency studies e.g., Myeki and Temoso (2019), Worthington and Lee (2008), Agasisti and Johnes, 2009
Postgraduates enrolled	Number of persons who are registered for a qualification which has an undergraduate qualification as an entrance requirement and with NQF exit levels 8, 9 and 10 (excluding 1st bachelor’s degrees with NQF exit level 8). It is measured in total headcount enrolment	This is also a commonly used input variable in the higher education literature (Witte and López-Torres (2017)
Academic staff	Refers to university employees who spend at least 50% of their official time on duty on teaching and/or research activities. This variable is measured in full time equivalent (FTE)—for instance employees who work full‐time at the institution for an entire year are taken as single full‐time equivalent staff units	This variable is included to reflect the basic input to the higher education production process (Moore et al., 2019 and Johnes, 2008)
Other cost (*South African Rands*)	Refers to the expenditure of the university in a given year measured in Rands. This includes operating expenses of the university minus labour costs. This expenditure is deflated using the Consumer Price Index (CPI) to obtain the real values	Other studies that include this are Agasisti and Salerno, 2007, Worthington and Lee (2008), Myeki and Temoso (2019)
*Drivers of productivity and efficiency growth*		
*Institutional type*	This is a proxy variable that accounts for university heterogeneity and assesses whether the historical reputation of the university matters for its performance i.e., does being historically advantaged or disadvantaged institutions impact the efficiency of universities (CHE, 2017, p. 134). Institutional type may highlight different responses to, and/or access and participation rates for learners	As highlighted by Witte and López-Torres (2017) this variable is commonly included in the literature and often produce mixed results
Graduation rates	Number of students who have graduated in a particular year, irrespective of the year of study, divided by the total number of students enrolled at the university, in that particular year. The results imply that success rates of universities significantly influence TFP growth, technical efficiency change and technological change	As shown by Witte and López-Torres (2017) this is also a common variable included as an output variable in the literature
Academic qualifications (% with PhDs)	Proportion of employees with doctoral degrees at least 50% of their official time on duty on teaching and/or research activities (CHE, 2019). This is a proxy for academic staff quality	Other studies that include this environmental variable includes Sav (2012b), Johnes and Yu (2008), Lee (2011)
Academic-student ratio	Number of academic full-time equivalent employees divided by the number of students who are registered at the university. As pointed out by Witte and López-Torres (2017) lower academic-student ratios may have a positive effect on students’ achievement because they can be more focussed on the students	This is one of the popular environmental variables used in the literature (Witte and López-Torres (2017)
Main source of income	Refers to the principal provider of income to fund the expenses in the university. We expect those universities that entirely depend on government funding to perform worse off than those which have diverse sources of funds (private tuition fees, donors, patents, etc.)	Other studies that include this in their model includes Lee (2011)
University location (province)	Location refers to the province where the main campus of the university is located. This variable is included to capture neighbourhood characteristics of where the university is located. According to Lee (2011) a positive effect of location to efficiency may suggest universities located in urbanised areas have more and better opportunities in research collaboration and appealing to both students and academics because of availability and quality of local amenities	In their systematic review, Witte and López-Torres (2017) found 16 papers that include location proxies in the study

*Source* Centre for Higher Education Transformation (CHET)- South Africa Higher Education Performance Indicators (2009–2016)

## Data and Sources

### Data Sources and Measurement Variables

The data for this study is obtained from the official website of South African Department of Higher Education and Training (DHET). There are 22 public universities (representing 85% of all universities in South Africa) in our sample which are analysed from 2009 to 2016, with a total of 176 observations. They are classified into traditional universities, comprehensive universities, and technology universities and are located across seven provinces of South Africa. Four universities (Sol Plaatje University, University of Mpumalanga, Mangosuthu University of Technology and Sefako Makgatho Health Science University) were excluded from the analysis due to their incomplete data for the observed period. Following previous studies on HEI efficiency and productivity (e.g., Carrington et al., 2018; Lee, 2011; Myeki & Temoso, 2019; Worthington & Lee, 2008), the input–output framework which reflects the university behaviour is modelled through a production function approach. This approach assumes universities utilise labour and non-labour inputs to produce outputs in the form of teaching and research outputs and grants (income) received (Lee, 2011).

Five output and five input variables are considered in the analysis. Output variables include number of undergraduates completed, postgraduates completed, doctorates completed, number of research output (publications) and total block grants (income received). Block grants represent income earned by institutions based on their research and teaching performance. There is no consensus in the literature on whether this variable should be treated as an output or input variable. For example, Carrington et al. (2018) and Nkohla et al. (2019) treated it as an input variable, whilst Flegg and Allen (2007) and Worthington and Lee (2008) treated it as an output variable. For this study we treated block grants (our proxy for teaching and research income) as an output variable because the grant is awarded to universities with a successful record in producing teaching and research outcomes. In terms of input variables, we included undergraduates enrolled, postgraduates enrolled, academic staff, support staff and other costs. Definitions of all variables used for the study are presented in Table 1, while summary statistics are given in Table 2.

**Table 2:** Summary statistics of inputs and outputs variables for the South African universities

Variable	All		Traditional		Comprehensive		Technology	
	Mean	SD	Mean	SD	Mean	SD	Mean	SD
Undergraduates Completed	5650	4893	4333	2741	7631	7767	6168	2995
Postgraduates completed	2729	3142	3313	2305	3111	4437	987	2155
Doctorates completed	90	87	139	85	61	70	18	16
Publications	602	561	908	562	427	436	137	82
Block grant (million Rands)	145	86	155	78	149	106	116	69
Undergraduates enrolled (FTE)	34,354	52,024	21,542	11,431	63,101	92,174	28,041	13,627
Postgraduates enrolled	6846	8654	8313	5299	8905	13799	1150	766
Academic staff (FTE)	1076	689	1,209	560	1101	988	752	328
Support Staff (FTE)	1428	821	1665	761	1377	987	969	463
Other costs (million Rands)	185	138	236	146	162	130	103	65

### Summary Statistics

Table 2 provides summary statistics for the South African universities data. The results show that South Africa has a diverse university sector. There are clear differences in university production inputs and outputs, ascribed to the differences in the mission focus of the three-university classification (see Sect. The South African University Sector). In terms of outputs, the sector’s average for undergraduate completions is 5650 students, with comprehensive universities (7631) and technology universities (6168) producing the largest number of graduates during the study period. Similarly, comprehensive universities enrol the largest number of undergraduates students followed by technology and traditional universities, respectively. Therefore, compared to the traditional universities, these universities (technology universities and comprehensive universities) can be seen as specialists on teaching output rather than research output.

The results show that comprehensive universities (63,101) enrolled as much as three times the number of undergraduate students enrolled by traditional universities (21,542), however, only produced twice as much of undergraduate completions. This may suggest that traditional universities are more efficient in undergraduate completions as compared to the other groups. However, these results may reflect the high dropout rates and low graduation rates in comprehensive and technology universities, which enrol a large proportion of students from disadvantaged backgrounds, as compared to the traditional universities (Cloete, 2016; DHET, 2018a, b).

Compared to the other groups, comprehensive universities received a large share of block grants during the study period. This may reflect the large funding received because of the large number and proportion of historically disadvantaged students enrolled in those universities. As pointed in Table 1, block grants are performance-based funding provided to South African HEI composed of four components that includes teaching input which is based on enrolments as well as teaching output, research output, and institutional factors based on size and proportion of historically disadvantaged students.

In terms of other output variables, as expected, traditional universities are dominant in all other outputs (postgraduates, PhD and master research completions, research outputs) followed by comprehensive universities. These results reflect the research-orientation of the traditional universities which are most favourably located and resourced and conduct most of the research in South Africa. With exception to the number of undergraduates enrolled, on average, traditional universities have a higher number of academic staff (FTE) and total expenditure.

## Results and Discussion

### South African Universities Productivity and Efficiency Levels

The Färe-Primont index estimations of TFP levels and their components for the 22 public universities in South Africa covering an 8-year period (2009–2016) are presented in Table 3, whilst the TFP results at the university level are provided in “Appendix A”. The average TFP level is 0.631, whilst TFP* (the maximum TFP that is possible using the available technology—combinations of inputs, in each period) is 0.80. On the other hand, average TFPE (i.e., TFP divided by TFP*) for the whole period is 0.787 which tells us that the productivity of the sector is 21% less than the maximum productivity that is possible using available technology for the period under review. In terms of TFPE components, OTE was 0.992 and OSME was 0.793 which implies that the entire productivity shortfall for the sector is due to scale and mix inefficiency, something that previous studies in South Africa have not investigated.

**Table 3:** Average annual total factor productivity (TFP) and efficiency levels, 2009 to 2016

Year	TFP	TFP*	TFPE = (OTE × OSME)	OTE	OSME = (OME × ROSE) = (OSE × RME)
2009	0.620	0.819	0.757	0.987	0.767
2010	0.581	0.820	0.708	0.982	0.721
2011	0.609	0.798	0.763	0.983	0.777
2012	0.617	0.741	0.832	0.995	0.836
2013	0.641	0.787	0.814	0.997	0.817
2014	0.652	0.796	0.820	1.000	0.820
2015	0.692	0.854	0.810	0.999	0.811
2016	0.641	0.804	0.798	0.996	0.801
Mean	0.631	0.802	0.787	0.992	0.793

TFP is TFP that is possible using the technology available in period t, whilst TFPE can be defined as ratios of measures of TFP i.e., is estimated by dividing TFP with TFP*. The other output-oriented measures OSE, OME, ROSE and RME are not reported here to save space

The same analysis can be applied to each period and at the university level to understand the sector’s performance for a given period. For example, in the last period of the study (2016), TFPE level was 0.802 which implies that the sector had a potential to increase its productivity by 19.8% in that year.

### South African Universities Productivity and Efficiency Over Time, 2009 to 2016

Table 4 reports a complete and coherent panel of estimates (inter-spatial and inter-temporal comparisons) of sectoral productivity change. The analysis assumes that technological change (TC) is the same for each group of universities in all sub-periods which implies that all universities have the same access to the same production possibility set. Consequently, any change in the production possibility set arising from changes in external factors including government intervention can affect all universities equally, either in terms of improvement or worsening of the production frontier (O’Donnell, 2011, 2012a, b).

**Table 4:** TFP and efficiency growth by university type, 2009 to 2016

Sub-period	University type	All universities	Traditional universities	Comprehensive universities	Technology universities
Whole period (2009 to 2016)	TFP	3.43%	4.85%	6.13%	1.41%
	TFPE	5.32%	6.77%	8.07%	3.27%
	TC	− 1.80%	− 1.80%	− 1.80%	− 1.80%
	OTE	0.83%	0.92%	1.15%	0.07%
	OSME	4.45%	5.80%	6.85%	3.19%
Sub-period 2009 to 2012	TFP	− 1.75%	0.02%	− 0.12%	− 1.39%
	TC	− 9.43%	− 9.43%	− 9.43%	− 9.43%
	TFPE	0.78%	10.43%	10.28%	8.88%
	OTE	− 0.48%	0.85%	1.06%	− 0.16%
	OSME	1.26%	9.50%	9.12%	9.05%
Sub-period 2012 to 2016	TFP	5.18%	4.84%	6.25%	2.79%
	TC	7.63%	7.63%	7.63%	7.63%
	TFPE	4.54%	− 3.66%	− 2.21%	− 5.62%
	OTE	1.31%	0.07%	0.09%	0.23%
	OSME	3.19%	− 3.70%	− 2.27%	− 5.86%

*TFP* indicates the average total factor productivity, *TFPE* is total factor productivity efficiency, *OTE* is output-oriented technical efficiency, *OSME* is the output-oriented scale and mix efficiency

The results show that the university sector increased its productivity by 3.43% for the study period (2009 to 2016). The best performing university group was the comprehensive universities with 6.13% increase in TFP followed by traditional universities with a 4.85% while the technology universities’ productivity slightly increased by 2.6%. The overall efficiency of the comprehensive universities was driven by OSME (6.85%) and OTE (1.15%). Similarly, TFP growth of traditional universities during the study period is due to strong OSME (5.80%) whilst contribution of OTE (0.92%) was minimal. Technology universities experienced the least TFP growth amongst the three groups, this was due to a stagnant OTE change. These results imply that over the study period, the universities were more productive through economically scaling and optimally producing a mix of outputs (research output, teaching output and block grants).

We further analysed productivity growth into two sub-periods for the purpose of evaluating the effect of policies and shocks. For the sub-period, 2009 to 2012, the productivity for the sector declined by 1.75% with both the technology and comprehensive universities experiencing a fall in productivity of 1.39% and 0.12%, respectively, whilst traditional universities experienced a small growth of 0.02%.

Several reasons can be attributed to these results. First, in 2009 a process of re-configuration took place which saw separation of basic and higher education (that is formation of DHET). As a result, much attention was dedicated to the structural and managerial capacity of universities and less on technological innovation which may have led to this slow growth. Second, in 2011 most universities were placed under administration and had to deal with reports from administrators and this may have diverted attention from focusing on improving productivity (DHET, 2016; HESA, 2015). Third, the sub-period was largely characterised by high dropout rates as well as a decline in state funding which may have had a negative contribution towards the productivity growth of universities. As shown in Table 4, the productivity decline during this period was due to the slowing down of technical change, whilst strong efficiency change in the traditional and comprehensive universities was not enough to uplift the overall sector productivity.

In the second sub-period (2012 to 2016), the sector experienced strong productivity growth of 5.18%. During this period, all university groups experienced an increase in productivity with comprehensive universities leading by 6.25% followed by traditional universities with 4.84% and then technology universities with 2.79%. The growth of the sector during this period was driven by a remarkable growth of technical change. A possible explanation for such a strong growth in technological change is the widespread use of information technology and electronic learning initiatives launched within the South African universities during that period (White Paper, 2014).

Furthermore, several policies and legislations enacted over this sub-period may have contributed to this productivity growth. These include the *Higher Education and Training Amendment Bill*, *Review of Funding Framework for Universities* and *Reporting Regulations for Public Higher Education Institutions*. They were envisaged to address some of the challenges that offset growth in the previous period including ageing academics and inequity in remuneration amongst academics. Consequently, during this period the higher education sector witnessed an increase in student participation rates as well as graduate outputs (except for PhDs). Equity in access also improved during this period, with black students accounting for the largest proportion (72%) of total headcount.

### How Does Historically Disadvantaged Universities' Performance Compare to the Historically Advantaged Universities?

The study also classified the sampled universities into historically advantaged and disadvantaged. This was done to assess whether the historically disadvantaged are catching-up with the historically advantaged universities. The historically disadvantaged universities comprised of 12 universities (FH, UWC, UZ, UL, WSU, UNIVEN, UNISA, CPUT, CUT, TUT, VUT and DUT) whereas the historically advantaged universities were 10 (UP, UCT, SU, WITS, RU, NWU, NMU, FS, UJ and UKZN). The former had an average TFP level of 0.823 over the study period, whilst the latter commanded an average TFP of 0.893. The findings support popular global university rankings which have consistently ranked traditionally advantaged universities as the best universities in both South Africa and the whole Africa (UniRank, 2021).

**Fig. 1:**
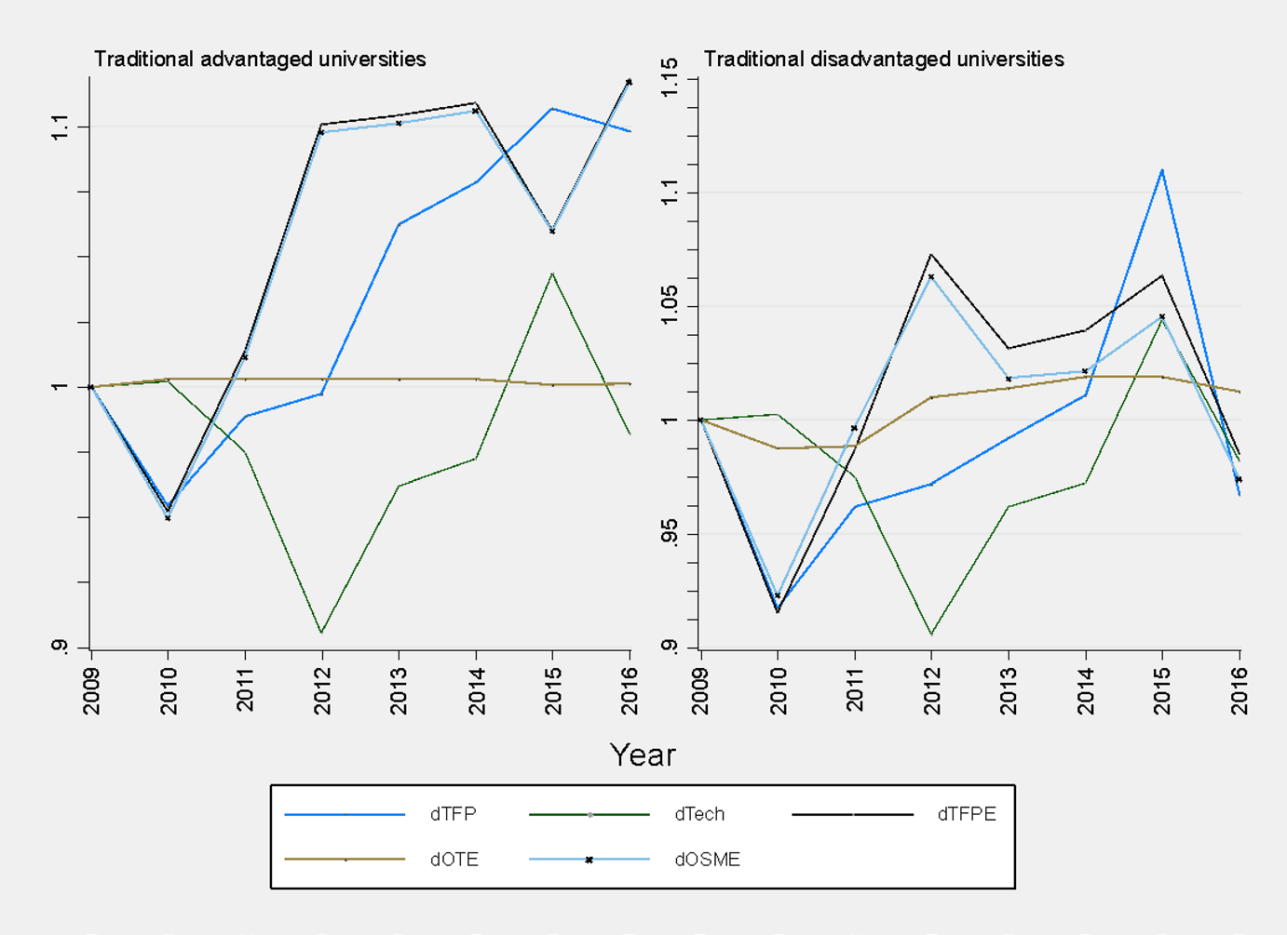
Cumulative TFP change and its components for traditional advantaged and disadvantaged universities, 2009 to 2016. Data source: Input and output data from Council for Higher Education report 2019

As shown in Fig. 1, it appears that a gap still exists between the two groups, with historically advantaged universities showing significant increase in TFP since 2010 as compared to historically disadvantaged universities. The results show that the TFP growth over the period was due to a strong growth in efficiency, particularly, scale-and mix efficiency (OSME). According to O’Donnell (2018, p. 382) an increase in OSME may reflect “how well managers have captured economies of scale and substitution (i.e., the benefits obtained by changing the scale of operations, the output mix, and the input mix)”. Hence, these results may reflect the nature of historically advantaged universities' ability to change their scale and output mix. Historically advantaged universities have better resource endowments in terms of infrastructure and funding and are more experienced in teaching and research and generate more research output and graduates (Myeki & Temoso, 2019). These universities are also highly selective in terms of student enrolments, choosing the best students, and they collaborate widely with industry and international research institutions.

The dichotomy in the performance of the aforesaid groups of universities (traditionally advantaged versus disadvantaged) is due to several factors. According to Van den Berg (2005), these factors include the quality of students enrolled, geographic location, financial health, infrastructural development, governance issues and social and political context within which the university operates. Other studies (e.g., CHE, 2019; Tjønneland, 2017) have discussed that research and teaching outputs, contact mode of delivery, personal quality of students and academics and other institutional factors contribute to disparities between historically advantaged and disadvantaged universities performance.

### Sources of TFP and Efficiency Changes for the South African Universities

Following the estimation of the TFP index, panel data regression models were used to assess the determinants of TFP and its components. We applied a Feasible Generalized Least Square (FGLS) model to examine the possible predictors and institutional factors influencing the HEI productivity. The results of the TFP and efficiency determinants are presented in Table 5. Apart from government funding (main source of university funding being government grants) which has a positive effect (but not statistically significant) on TFP, most variables (graduation rates, quality of academics i.e., academics with PhD and academic-student ratio) influenced TFP positively.

**Table 5:** Sources of TFP and efficiency changes for the South African Universities, 2009 to 2016

Variable	dtfp(1)	dtfpe(2)	dote(3)	dosme(4)
ln_gradrate	0.843***	0.829***	0.00404	0.769***
	(6.16)	(6.02)	(0.17)	(5.34)
ln_acadPhD	0.168***	0.174***	0.00970	0.138***
	(8.08)	(8.26)	(1.78)	(5.96)
ln_acadstu	0.239***	0.239***	0.00206	0.227***
	(9.85)	(9.79)	(0.52)	(9.18)
ln_stufees	0.0374	0.0416	0.00561	0.0262
	(0.98)	(1.08)	(0.83)	(0.63)
ln_govfund	0.0113	0.0139	− 0.00467	0.0283
	(0.31)	(0.38)	(− 0.74)	(0.76)
Institutional Type (base = *Traditional*)				
*Comprehensive*	0.0317	0.0340	− 0.00171	0.0371
	(1.55)	(1.65)	(− 0.38)	(1.81)
*Technology*	0.0136	0.0164	0.00966	− 0.0155
	(0.50)	(0.60)	(1.63)	(− 0.57)
Province (base = *Western Cape*)				
KwaZulu Natal	0.128***	0.131***	0.00241	0.127***
	(5.58)	(5.61)	(0.63)	(5.26)
*Eastern Cape*	− 0.0902***	− 0.0918***	− 0.00112	− 0.0870***
	(− 4.28)	(− 4.32)	(− 0.31)	(− 4.07)
*Free State*	0.0601*	0.0580*	− 0.000497	0.0560*
	(2.47)	(2.34)	(− 0.11)	(2.41)
*Gauteng*	0.00568	0.00341	0.000462	− 0.00430
	(0.27)	(0.16)	(0.15)	(− 0.20)
*Limpopo*	0.0203	0.0186	0.00383	0.0106
	(0.59)	(0.53)	(0.68)	(0.30)
*North West*	− 0.00822	− 0.00699	0.000647	− 0.00406
	(− 0.38)	(− 0.32)	(0.24)	(− 0.19)
Year (base = 2009)				
2010	− 0.0626***	− 0.0648***	− 0.000189	− 0.0636***
	(− 3.48)	(− 3.55)	(− 0.07)	(0.23)
2011	− 0.0412*	− 0.0212	− 0.000619	0.000526
	(− 2.24)	(− 1.13)	(− 0.23)	(0.27)
2012	− 0.0541**	0.0318	− 0.000187	0.0000147
	(− 2.90)	(1.68)	(− 0.07)	(0.01)
2013	− 0.0396*	− 0.00605	− 0.000920	− 0.000141
	(− 2.01)	(− 0.30)	(− 0.34)	(− 0.07)
2014	− 0.0416*	− 0.0180	− 0.00167	− 0.000553
	(− 2.05)	(− 0.88)	(− 0.57)	(− 0.26)
2015	− 0.0156	− 0.0558**	− 0.00217	− 0.00197
	(− 0.73)	(− 2.59)	(− 0.72)	(− 0.85)
2016	− 0.0788***	− 0.0639**	− 0.00312	− 0.00137
	(− 3.81)	(− 3.05)	(− 1.03)	(− 0.61)
_constant	0.562***	0.573***	1.004***	1.001***
	(5.63)	(5.67)	(55.68)	(85.63)
No. of obs	176	176	176	176

Robust standard errors in parentheses. *ln_gradrate* is log values of graduate rates; *ln_acadPhD* is log values of academic staff with a doctorate degree; *ln_acadstu* is the log values of academic to student ratio; *ln_stufees* is log values of share of student paying fees; *ln_govfund* is the log values of share of income from government funding

*Significant at the 10% level

**Significant at the 5% level

***Significant at the 1% level

Completion (graduation) rates (*ln_gradrate*) which is a proxy for a university's success and academic reputation was found to have a positive and significant effect on productivity (TFP), efficiency (TFPE) and OSME of the university. The results imply that those universities with high graduation rates tend to be more efficient and productive. This finding corroborates Sav (2012a) who found a positive relationship between graduation rates and efficiency of colleges in the United States of America. Therefore, part of the effort to increase graduation rate in the context of higher education in South Africa would need to consider measures for reducing student dropout rates.

The proportion of academics with doctoral degrees (*ln_acadPhD*), a proxy of academic staff quality, has a positive and statistically significant effect on TFP, TFPE and OSME. The results suggest that having a high proportion of PhD qualified academic staff positively contributes to an increase in productivity. Existent literature has reported mixed findings on this variable. For instance, Abbott and Doucouliagos (2009) found that higher proportions of academic professorial level are associated with higher levels of inefficiency due to being stuck in administration workload and also claiming that it just happens that these were poor staff seeking promotion to escape academic work and then interfere with the work of others. On the other hand, Sav (2013) confirms our finding. This implies that one possible avenue in which universities could uplift their productivity is through increasing the proportion of PhD qualified academic staff. This is in line with the *National Development Plan 2030 Strategy* which aims to achieve 75% PhD qualified academic staff across the sector by 2030 (NDP, 2011).

The number of academic full-time equivalent employees divided by the number of students who are registered at the university (*ln_acadstu*) positively and significantly influences TFP, TFPE and OSME. Our findings indicate that a high academic-student ratio positively improves productivity. The positive effect of this variable on OSME implies that academics with a small number of students can contribute to both teaching and research. The findings were earlier discovered by Kao and Hung (2008), Johnes and Yu (2008) and confirmed 4 years later by Sav (2012b) while analysing cost efficiency of public and private research universities in the United States of America. In the South Africa HEI context, Marire (2017) also found a positive effect of this variable on cost efficiency of HEI for the period 2009 to 2013.

The location (*province*) of the university is negative and statistically significant for the Eastern Cape province which suggests that, on average, universities located in this province are less productive than the rest of the other universities. Previous studies, for instance, Kempkes and Pohl (2008) used the location variable to account for efficiency differences between Eastern and Western Germany universities. Their findings reveal that universities located in the most remote (East Germany) areas are inefficient and less productive as compared to those in more developed West Germany. These results are likely to reflect place specific features that are not captured in the model such as level of economic development (urbanisation) as well as availability and quality of local amenities. For example, to a large extent Eastern Cape have semi-urban-based university campuses. In addition, most universities in this province fall under the category of traditionally disadvantaged universities and are more likely to be affected by the recent decline in government grants. Other environmental variables such as institutional type, student fees and government funding were not statistically significant, hence are not discussed.

## Summary, Conclusions and Policy Implications

This study estimated productivity and efficiency growth of 22 public universities in South Africa using a recently developed TFP method (Färe-Primont index) covering an 8-year period (2009–2016). This estimation was based on three university classifications (technology universities, traditional universities, and comprehensive universities) and two historical groupings (historically advantaged and historically disadvantaged).

The Färe-Primont TFP index results indicate that the average TFP of universities for the study period was 0.631, led by historically advantaged universities (Universities of Pretoria, Johannesburg, North West, and Stellenbosch), whilst historically disadvantaged universities (Universities of Limpopo, and Walter Sisulu) had lower average TFP. This leads to our first policy recommendation that the DHET should increase effort to improve the underutilized capacity through training and education of the academics including more incentives particularly for the historically disadvantaged universities.

In terms of university types, over the study period, the comprehensive universities achieved the largest productivity growth (6.13%) followed by traditional universities (4.85%), and technology universities by 1.41%. Both the comprehensive and traditional universities’ efficiency growth were driven by scale and mix efficiency change whilst the contribution of technical efficiency change was limited. On the other hand, across the three groups of universities, the technological change was negative. The second recommendation is that the DHET and other HEI-related stakeholders should increase their investment into research and development as well as adoption of teaching and research innovations to boost technical change in order to uplift the productivity of the university sector.

Through panel data regression analysis, the study has identified factors affecting the productivity and efficiency growth of universities. The results show that the productivity and efficiency growth of universities is positively affected by the university graduation rates i.e., universities with highest graduation rates are relatively more productive than others. We also found that quality of academics matters for university performance. That is, having more PhD qualified academics increases a university's productivity. The results suggest that one possible avenue in which universities could uplift their productivity is through increasing the proportion of PhD qualified academic staff.

Therefore, our third policy recommendation is that HEI stakeholders should strive to reduce the dropout rates by providing adequate student funding, improved student accommodation and better well-being for students which also includes mental health related issues. Furthermore, the universities should improve their recruitment of staff by hiring PhD qualified academics and at the same time encourage those already in the system to improve their qualifications to the doctoral level. Doing so, would also contribute towards the achievement of the *National Development Plan 2030 Strategy* (NDP, 2011) goals of increasing the proportion of PhD qualified academic staff at South African universities and training at least 100 PhD graduates per million people per annum by 2030.

Universities with high academic-student ratios achieved higher productivity and efficiency growths. This finding measures the quality of output from the country’s universities. Recently, there has been a general scepticism about the quality of HEIs due to lack of skills despite the increasing number of certificated persons from these HEIs. Our fourth recommendation is that university admission policies must be closely aligned to the size of the academic staff.

## Appendix A: Indexes of changes in total factor productivity in South African universities (base: CPUT 2009 = 1)

**Table:**

University	2009	2010	2011	2012	2013	2014	2015	2016	Average	Rank
UNIVEN	0.959	1.098	1.068	0.993	0.963	1.065	1.103	0.940	1.024	1
UP	1.031	0.903	0.965	0.944	0.998	1.066	1.092	1.076	1.009	2
DUT	1.038	0.870	0.879	0.860	0.950	0.974	1.130	0.976	0.960	3
NWU	1.034	0.953	0.956	0.884	0.930	0.897	0.976	1.032	0.958	4
KZN	0.781	0.812	0.825	0.924	1.054	1.062	1.091	1.013	0.945	5
FS	0.843	0.854	0.911	0.913	0.929	0.960	1.066	1.033	0.939	6
UNISA	1.096	0.751	0.917	0.921	0.997	0.935	0.974	0.913	0.938	7
SU	0.928	0.820	0.874	0.910	0.933	0.955	0.982	0.980	0.923	8
UJ	0.759	0.773	0.789	0.832	0.985	0.978	1.091	1.064	0.909	9
UWC	0.870	0.869	0.888	0.865	0.920	0.943	0.937	0.862	0.894	10
NMU	0.916	0.874	0.951	0.905	0.906	0.899	0.894	0.803	0.894	11
TUT	0.996	0.811	0.812	0.867	0.879	0.870	0.950	0.809	0.874	12
UCT	0.877	0.848	0.851	0.826	0.851	0.836	0.799	0.743	0.829	13
RU	0.795	0.750	0.719	0.808	0.844	0.874	0.879	0.892	0.820	14
CPUT	1.000	0.843	0.820	0.801	0.784	0.775	0.807	0.650	0.810	15
CUT	0.860	0.871	0.809	0.800	0.811	0.737	0.771	0.791	0.806	16
FH	0.580	0.762	0.808	0.840	0.844	0.865	0.874	0.805	0.797	17
UZ	0.690	0.602	0.674	0.769	0.793	0.845	0.943	0.865	0.773	18
UL	0.538	0.589	0.606	0.669	0.632	0.743	1.144	0.803	0.716	19
WITS	0.659	0.642	0.683	0.652	0.728	0.773	0.671	0.834	0.705	20
VUT	0.743	0.598	0.656	0.673	0.657	0.655	0.678	0.605	0.658	21
WSU	0.610	0.494	0.662	0.641	0.672	0.679	0.769	0.634	0.645	22

*UCT* University of Cape Town, *FH* Fort Hare, *FS* Free State, *KZN* Kwa Zulu Natal, *UL* University of Limpopo, *NWU* North West University, *UP* University of Pretoria, *RU* Rhodes University, *SU* Stellenbosch University, *UWC* University of Western Cape, *WITS* Witwatersrand University, *UJ* University of Johannesburg, *NMU* Nelson Mandela University, *UNISA* University of South Africa, *UNIVEN* University of Venda, *WSU* Walter Sisulu University, *UZ* University of Zululand, *CPUT* Cape Peninsula University of Technology, *CUT* Central University of Technology, *DUT* Durban University of Technology, *TUT* Tshwane University of Technology, *VUT* Vaal University of Technology

## Footnotes

1 The block grants are linked to performance and have four components: (1) teaching input which is based on enrolments, (2) teaching output which is based on graduations, (3) research output, based on approved publications and research students’ graduations, and (4) institutional factors based on size and proportion of historically disadvantaged students.

## References

[CR1] Abbott M, Doucouliagos C (2009). Competition and efficiency: Overseas students and technical efficiency in Australian and New Zealand universities. Education Economics.

[CR2] Agasisti T (2017). Management of higher education institutions and the evaluation of their efficiency and performance. Tertiary Education and Management.

[CR4] Agasisti T, Johnes G (2009). Beyond frontiers: Comparing the efficiency of higher education decision-making units across more than one country. Education Economics.

[CR3] Agasisti T, Salerno C (2007). Assessing the cost efficiency of Italian Universities. Education Economics.

[CR5] Akoojee S, Nkomo M (2007). Access and quality in South African higher education: The twin challenges of transformation. South African Journal of Higher Education.

[CR6] Badat, S. (2008). Redressing the colonial/apartheid legacy: Social equity, redress and higher education admissions in democratic South Africa. In *Paper presented at the conference on affirmative action in higher education in India, the United States and South Africa, New Delhi, India, 19–21 March*.

[CR7] Blackburn V, Brennan S, Ruggiero J (2014). Nonparametric estimation of educational production and costs using data envelopment analysis.

[CR8] Boughey C (2003). From equity to efficiency: Access to higher education in South Africa. Arts and Humanities in Higher Education.

[CR10] Bunting, I. (2006). The higher education landscape under apartheid. In *Transformation in higher education* (pp. 35–52). Springer.

[CR11] Cantele S, Guerrini A, Campedelli B (2016). Efficiency of Italian Universities: The effect of controllable and non-controllable environmental and operational variables. International Journal of Public Policy.

[CR12] Carrington R, O'Donnell C, Rao DS (2018). Australian university productivity growth and public funding revisited. Studies in Higher Education.

[CR13] Cloete N (2016). For sustainable funding and fees, the undergraduate system in South Africa must be restructured. South African Journal of Science.

[CR14] Council on Higher Education. (2004). *Higher education in the first decade of democracy*. Council on Higher Education. Retrieved from http://www.che.ac.za/.

[CR15] Council of Higher Education. (2007). *Review of higher education in South Africa: Selected themes. Pretoria, South Africa*. Retrieved from http://www.che.ac.za/.

[CR16] Council on Higher Education. (2016). *South African higher education reviewed: Two decades of democracy*. CHE. Retrieved from http://www.che.ac.za/.

[CR17] Council on Higher Education. (2017). *Review of higher education in South Africa: Selected themes*. Council on Higher Education. Retrieved from http://www.che.ac.za/.

[CR18] Council on Higher Education. (2019). *Vital stats—public higher education 2017*. Council on Higher Education. Retrieved from http://www.che.ac.za/.

[CR19] De Fraja G, Valbonesi P (2012). The design of the university system. Journal of Public Economics.

[CR22] Department of Higher Education and Training. (2012). *Green paper for post-school education and training*. DHET. Retrieved from www.dhet.gov.za.

[CR24] Department of Higher Education and Training. (2016). *Statistics on post-school education and training in South Africa*. DHET. Retrieved from www.dhet.gov.za.

[CR25] Department of Higher Education and Training. (2018a). *Ministerial statement on university funding: 2019/20 and 2020/21*. DHET. Retrieved from www.dhet.gov.za.

[CR26] Department of Higher Education and Training. (2018b). *Statistics on post-school education and training in South Africa*. DHET. Retrieved from www.dhet.gov.za.

[CR27] Department of Higher Education and Training. (2019). *Statistics on post-school education and training in South Africa*. DHET. Retrieved from www.dhet.gov.za.

[CR28] Edvardsen DF, Førsund FR, Kittelsen SA (2017). Productivity development of Norwegian institutions of higher education 2004–2013. Journal of the Operational Research Society.

[CR29] Flegg AT, Allen DO (2007). Does expansion cause congestion? The case of the older British universities, 1994–2004. Education Economics.

[CR31] Glass JC, McKillop DG, O'Rourke G (1998). A cost indirect evaluation of productivity change in UK universities. Journal of Productivity Analysis.

[CR32] Govinder KS, Zondo NP, Makgoba MW (2013). A new look at demographic transformation for universities in South Africa. South African Journal of Science.

[CR33] Higher Education South Africa (HESA). (2015). *Higher education summit: Reflections on higher education transformation*. HESA. Retrieved from https://www.dhet.gov.za/summit.

[CR34] Johnes J (2008). Efficiency and productivity change in the English higher education sector from 1996/97 to 2004/5. The Manchester School.

[CR35] Johnes J, Yu L (2008). Measuring the research performance of Chinese higher education institutions using data envelopment analysis. China Economic Review.

[CR36] Kao C, Hung H-T (2008). Efficiency analysis of university departments: An empirical study. Omega.

[CR37] Kempkes G, Pohl C (2008). Do institutions matter for university cost efficiency? Evidence from Germany. Cesifo Economic Studies.

[CR38] Kuah CT, Wong KY (2011). Efficiency assessment of universities through data envelopment analysis. Procedia Computer Science.

[CR39] Lee BL (2011). Efficiency of research performance of Australian universities: A reappraisal using a bootstrap truncated regression approach. Economic Analysis & Policy.

[CR40] Mabokela RO, Mlambo YA (2017). Access and equity and South African higher education: A review of policies after 20 years of democracy. Comparative Education Review.

[CR41] Marire J (2017). Are South African public universities economically efficient? Reflection amidst higher education crisis. South African Journal of Higher Education.

[CR42] Miles PC, Peterson M, Miles G, Bement D (2018). Higher education: Exploring productivity over time. Journal of Applied Research in Higher Education.

[CR43] Moloi KC, Mkwanazi TS, Bojabotseha TP (2014). Higher education in South Africa at the crossroads. Mediterranean Journal of Social Sciences.

[CR44] Moore K, Coates H, Croucher G (2019). Investigating applications of university productivity measurement models using Australian data. Studies in Higher Education.

[CR45] Myeki LW, Temoso O (2019). Efficiency assessment of public universities in South Africa, 2009–2013: Panel data evidence. South African Journal of Higher Education.

[CR47] National Planning Commision. (NPC). (2011). *National development plan 2030: Our future—make it work*. National Planning Commission. Retrieved from https://www.nationalplanningcommission.org.za.

[CR48] Nkohla, V., Munacinga, S., Ncwadi, R., & Marwa, N. (2019). A non-parametric assessment of efficiency of South African public universities. The biennial conference of Economic Society of South Africa, Johannesburg.

[CR49] O’Donnell CJ (2011). DPIN 3.0. A program for decomposing productivity index numbers.

[CR51] O’Donnell CJ (2012). An aggregate quantity framework for measuring and decomposing productivity change. Journal of Productivity Analysis.

[CR50] O’Donnell CJ (2012). Nonparametric estimates of the components of productivity and profitability change in US Agriculture. American Journal of Agricultural Economics.

[CR52] O’Donnell, C. J. (2017). Estimating total factor productivity change when no price or value-share data are available. In *Centre for efficiency and productivity analysis working papers, University of Queensland*.

[CR53] O'Donnell CJ (2018). Productivity and efficiency analysis.

[CR54] OECD (2019). Education at a glance 2019: OECD indicators.

[CR55] Rahman S, Salim R (2013). Six decades of total factor productivity change and sources of growth in Bangladesh agriculture (1948–2008). Journal of Agricultural Economics.

[CR58] Sav GT (2012). For-profit college entry and cost efficiency: Stochastic frontier estimates vs two-year public and non-profit colleges. International Business Research.

[CR59] Sav GT (2012). Stochastic cost inefficiency estimates and rankings of public and private research and doctoral granting universities. Journal of Knowledge Management, Economics, and Information Technology.

[CR61] Sav GT (2013). Private philanthropy in financing public universities: Fundraising stochastic frontier and efficiency evaluations. International Research Journal of Finance and Economics.

[CR63] Taylor B, Harris G (2004). Relative efficiency among South African universities: A data envelopment analysis. Higher Education.

[CR64] Temoso O, Villano R, Hadley D (2015). Agricultural productivity, efficiency and growth in a semi-arid country: A case study of Botswana. African Journal of Agricultural and Resource Economics.

[CR65] Tjønneland, E. N. (2017). Crisis at South Africa’s universities—what are the implications for future cooperation with Norway? Retrieved from https://www.cmi.no/publications.

[CR66] Tran CDT, Villano RA (2017). Input rigidities and performance of Vietnamese Universities. Asian Economic Journal.

[CR67] UniRank. (2021). *Top 200 universities in Africa: 2021 African University ranking*. Retrieved from https://www.4icu.org/top-universities-africa/.

[CR100] Van den Berg MN, Hofman WHA (2005). Student success in university education: A multi-measurement study of the impact of student and faculty factors on study progress. Higher Education.

[CR69] White Paper. (2014). *White paper for post-school education and training: Building an expanded, effective and integrated post-school system*. DHET. Retrieved from https://www.gov.za.

[CR70] Widodo W, Salim R, Bloch H (2014). Agglomeration economies and productivity growth in manufacturing industry: Empirical evidence from Indonesia. Economic Record.

[CR71] Witte KD, López-Torres L (2017). Efficiency in education: A review of literature and a way forward. Journal of the Operational Research Society.

[CR72] Worthington AC, Lee BL (2008). Efficiency, technology and productivity change in Australian universities, 1998–2003. Economics of Education Review.

